# The degeneration of uncovertebral joints on CT assessment and its association with postoperative heterotopic ossification in one- or two-level artificial cervical arthroplasty

**DOI:** 10.3389/fsurg.2026.1911386

**Published:** 2026-10-05

**Authors:** Yi Yang, Weiyang Zhong

**Affiliations:** 1 Department of Orthopaedic Surgery, The First Affiliated Hospital of Chongqing Medical University, Chongqing, China; 2 Department of Orthopaedics, Chongqing Municipal Health Commission Key Laboratory of Musculoskeletal Regeneration and Translational Medicine, Chongqing, China; 3 Department of Orthopaedics, Orthopaedic Research Laboratory of Chongqing Medical University, Chongqing, China

**Keywords:** artificial cervical arthroplasty, CT assessment, heterotopic ossification, segmental range of motion, uncovertebral joint degeneration

## Abstract

**Background:**

Artificial cervical arthroplasty (ACR) preserves segmental mobility and reduces adjacent segment degenerative disease risk, yet postoperative heterotopic ossification (HO) impairs prosthetic function. Preoperative uncovertebral joint degeneration (UJD) is hypothesized to relate to HO. This study evaluated UJD on computed tomography (CT) and explored its association with HO after one- or two-level ACR, adjusting for confounders.

**Methods:**

Forty patients undergoing one- or two-level ACR with a ≥1-year follow-up (2013–2019) were enrolled. UJD was graded using a modified Kellgren-Lawrence (K–L) CT system; final follow-up HO was assessed on radiographs using the McAfee classification. Generalized estimating equations (GEE) were used to address intrapatient clustering and confounders. Binary and ordinal logistic GEE, including proportional odds testing and sensitivity analyses, assessed independent UJD–HO associations. Stratified Spearman correlations were applied only for exploratory subgroup analyses.

**Results:**

Overall HO incidence was 92.0%, with four segments at McAfee grade 0. After adjusting for age, sex, follow-up duration, operative level number and preoperative range of mobility, GEE showed non-significant trends linking higher-grade UJD to greater HO occurrence [adjusted odds ratio (aOR) = 12.561, 95% CI 0.885–178.247, *P* = 0.061] and severity (aOR = 1.493, 95% CI 0.863–2.585, *P* = 0.152). Partial proportional odds sensitivity analyses showed consistent results. Severe HO distribution imbalance and a scarcity of HO-negative segments led to unstable effect estimates and wide confidence intervals.

**Conclusions:**

After adjusting for intrapatient clustering and confounders, preoperative UJD was not an independent predictor of HO onset or progression after ACR. Despite a positive trend, the high HO incidence caused a ceiling effect and limited statistical power, and thus, UJD's predictive value could not be confirmed. This CT-based modified K–L grading system reliably quantifies UJD and supports preoperative imaging evaluation for ACR candidates.

## 1 Introduction

Anterior cervical discectomy and fusion (ACDF) is an effective procedure for cervical radiculopathy and myelopathy, yielding reliable neural decompression and favorable cervical alignment (1, 2). However, ACDF inevitably causes operative segment motion loss and subsequent adjacent segment degenerative disease (ASDD). Artificial cervical arthroplasty (ACR) has been widely used to preserve segmental mobility and reduce the risk of ASDD (3). Although long-term studies have verified satisfactory clinical efficacy of ACR, postoperative heterotopic ossification (HO) remains the primary complication that impairs segmental range of mobility (ROM) and affects surgical decision-making (3–5). HO substantially restricts prosthetic and segmental mobility. Severe HO can induce neurological compression and may necessitate revision surgery.

Various intraoperative and perioperative protocols have been proposed to prevent HO formation. The uncovertebral joint (UJ), a critical structure limiting cervical lateral translation, is considered a potential contributor to HO pathogenesis. Biomechanically, degeneration of the UJ can alter joint mechanics, leading to increased friction and abnormal loading of adjacent structures. Moreover, uncovertebral joint degeneration (UJD) induces intra-articular inflammation that may predispose periprosthetic tissues to heterotopic bone formation (6–9). Currently, the quantitative evaluation of UJD mainly relies on plain radiographs (10), and unified bony grading criteria are still lacking. Compared with radiography and magnetic resonance imaging, computed tomography (CT) provides superior visualization of bony degenerative changes (11). Notably, few studies have explored the segment-specific association between UJD and postoperative HO.

The primary hypothesis was that higher CT-based preoperative UJD grades would correlate with more severe postoperative HO in patients undergoing single- or two-level ACR. The secondary objective was to verify the reliability of a modified CT-based Kellgren–Lawrence (K–L) grading system for UJD evaluation. This study aimed to fill the methodological gap in standardized UJD bony evaluation, emphasize the segmental heterogeneity of the cervical spine, and explore the association between UJD and HO. All findings are exploratory and limited to correlational observations, with no causal relationships confirmed.

## 2 Material and methods

### 2.1 Study design

Patients who underwent ACR from January 2013 to December 2019 were retrospectively reviewed. Inclusion criteria were as follows: (1) a diagnosis of single- or two-level cervical radiculopathy or myelopathy refractory to conservative treatment; (2) patients who consented to ACR surgery; (3) a minimum follow-up (FU) duration of 1 year; and (4) complete preoperative CT imaging data. Exclusion criteria included cervical ossification of the posterior longitudinal ligament, prior cervical spine surgery, cervical fracture, spinal tumor, spinal infection, segmental instability, severe intervertebral height loss, and age over 60 years.

Patients with grade 3 UJD were still considered eligible for ACR if they met the following conditions: (1) intractable cervical radiculopathy or myelopathy unresponsive to conservative management; (2) high-grade degeneration limited to the surgical segments; (3) absence of segmental instability contraindicating arthroplasty; and (4) age younger than 60 years without severe degenerative changes at adjacent segments. Although the modified K–L grading system covers grade 0 to grade 4, no patients with grade 4 UJD were included in the present cohort, and all enrolled segments exhibited UJD grades ranging from 0 to 3.

ACR was performed in these patients to preserve segmental mobility and reduce the risk of adjacent segment degeneration. Compared with ACDF, arthrodesis-induced motion loss and increased mechanical stress on adjacent segments can be avoided with ACR, which provides theoretical advantages in maintaining long-term cervical spinal biomechanics (12, 13).

### 2.2 Efficacy evaluation

All patients underwent preoperative CT scanning (Siemens, Germany) with a 0.5 mm slice thickness covering the C1–T2 levels. Three-dimensional reconstruction was performed using the picture archiving and communication system (PACS). Given the absence of standardized bony grading criteria for UJD, a modified CT-based K–L classification was applied in this study. This modified grading system incorporates coronal and axial CT planes to precisely evaluate uncovertebral joint osteophytes and joint space narrowing (14). Grading criteria were defined as follows: Grade 0, normal and symmetric uncovertebral joints; Grade 1, mild joint space narrowing or subtle osteophyte formation; Grade 2, measurable joint narrowing or osteophytes confined within the intervertebral level; Grade 3, definite joint narrowing or osteophytes extending beyond the intervertebral level; Grade 4, osteophytic articulation or complete uncinate joint fusion (10, 11) (Figure 1). Postoperative HO was graded via the McAfee classification on final follow-up radiographs (15) (Figure 2). Grades 1–2 were defined as low-grade HO, whereas Grades 3–4 were defined as high-grade HO. Segmental ROM was measured on full flexion–extension lateral radiographs.

**Figure 1:**
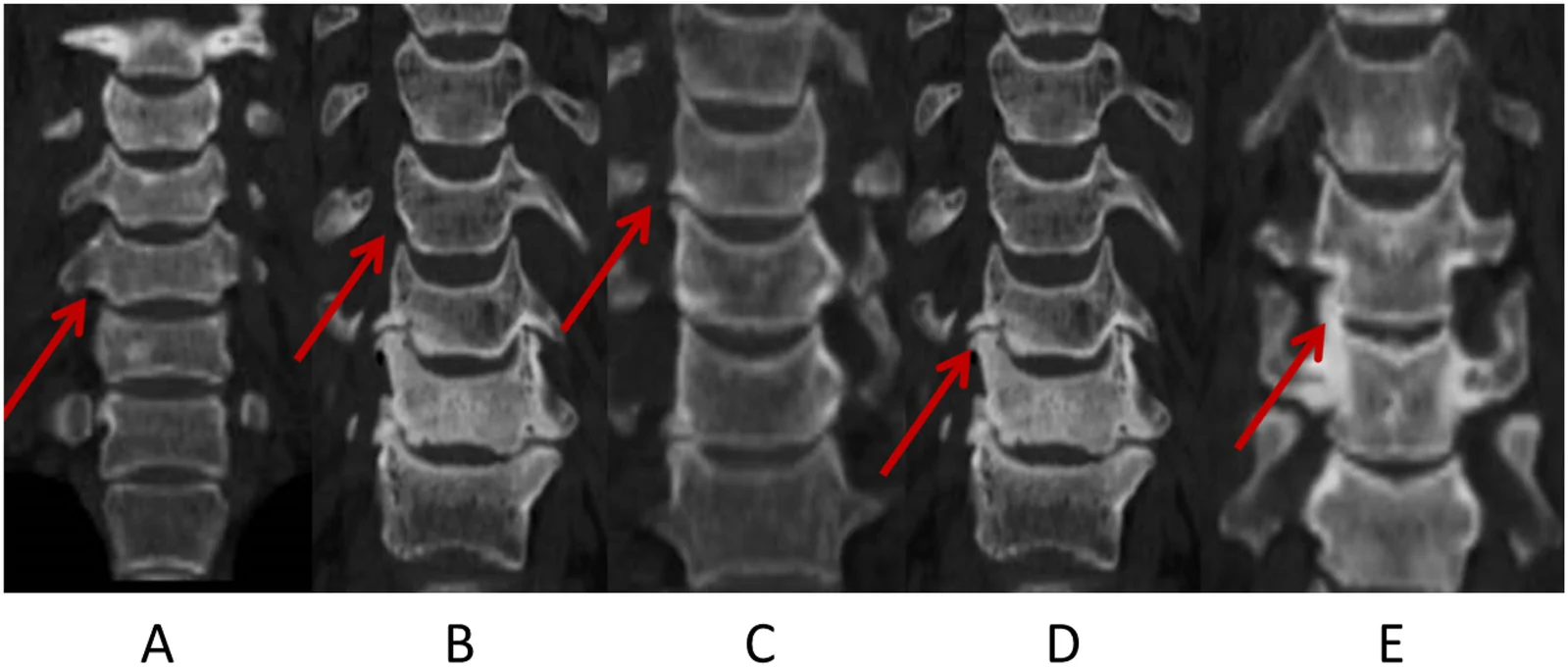
Modified CT-based Kellgren–Lawrence classification for UJD. Representative coronal and axial CT images of UJD Grades 0–4 are shown. **(A)** Grade 0: the joints were normal, bilateral, and symmetrical. **(B)** Grade 1: Mild space narrowing or osteophyte formation. **(C)** Grade 2: Joint space narrowing or osteophyte formation with osteophytes not exceeding the intervertebral level. **(D)** Grade 3: Definite joint space narrowing or osteophyte formation with osteophyte formation exceeding the intervertebral level. **(E)** Grade 4: Osteophytes at the articulation or uncinate joint fusion.

**Figure 2:**
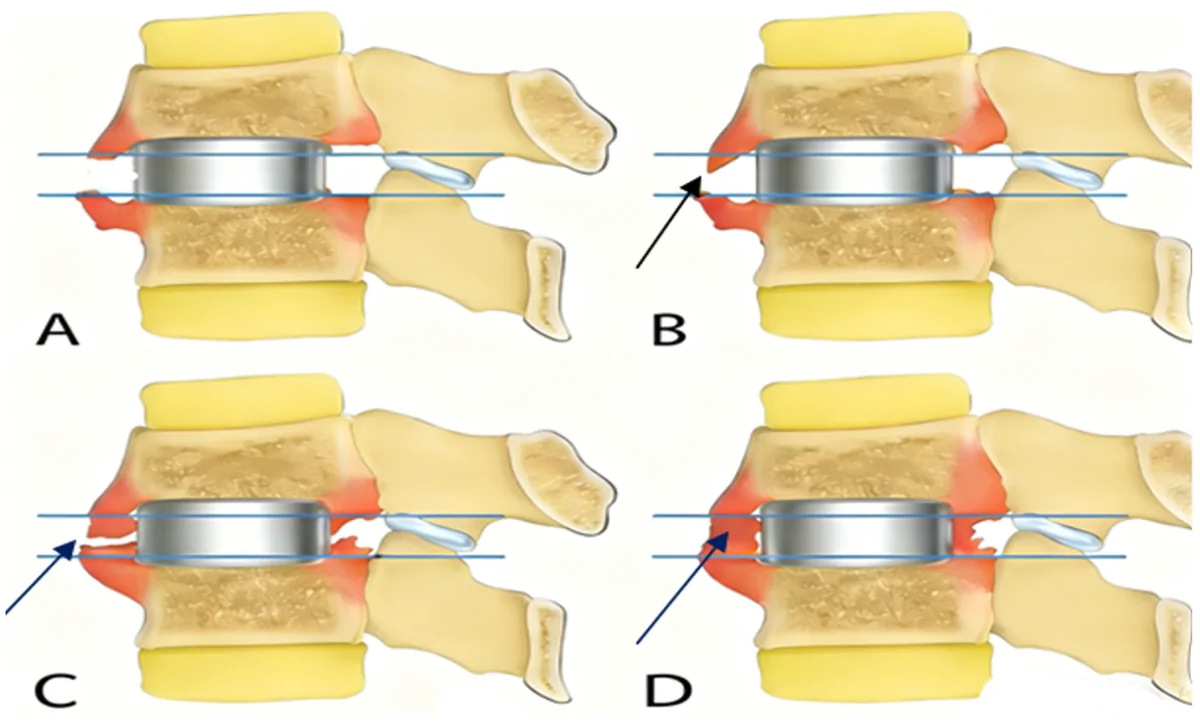
McAfee classification for HO. **(A)** Grade 1, bone formation detectable anterior to the vertebral body but not between the planes formed by the two vertebral endplates and not blocking motion. **(B)** Grade 2, bone present between the planes formed by the vertebral endplates but not blocking motion; **(C)** Grade 3, bone present between the planes formed by the vertebral endplates and restricting motion; **(D)** Grade 4, complete bony ankylosis across the treated segment prevent motion through the prosthesis.

All radiographic assessments were independently performed by a senior spine surgeon (10 years of experience) and a specialized radiologist (8 years of experience). Interobserver agreement for CT-based UJD and HO grading was evaluated using Cohen's Kappa coefficient, while intraobserver reliability was tested using the intraclass correlation coefficient (ICC). For intraobserver testing, all images were randomly reordered and reassessed by the two observers after a 4-week interval, with both raters blinded to prior results. Discrepancies were resolved through consensus discussion. Persistent disagreements were adjudicated by an independent senior reviewer.

### 2.3 Statistical analysis

All statistical analyses were performed using SAS software. A total of 40 patients involving 50 operative segments were enrolled, including 30 single-level and 10 two-level ACR cases. Unique patient clustering IDs were established, and no missing data existed for age, sex, follow-up duration, operative level number, UJD grade, McAfee HO grade, and segmental ROM. Given that multiple segments were derived from the same patient, segment-level observations were statistically non-independent. Generalized estimating equations (GEE) with an exchangeable working correlation matrix and robust standard errors were applied to control for intrapatient clustering.

Two core GEE models were constructed to assess the primary hypothesis. The binary logistic GEE model was used to analyze the association between preoperative UJD grade and HO occurrence, with McAfee grade 0 defined as non-HO and grades 1–4 defined as HO-positive. The ordinal cumulative logistic GEE model was fitted to evaluate the relationship between UJD grade and ordinal HO severity (McAfee 0–4). Both models were adjusted for age, sex, follow-up duration, number of operative levels, and preoperative ROM.

Prior to ordinal GEE modeling, threshold-specific GEE tests were conducted to verify the proportional odds assumption. When the global assumption was violated, the conventional ordinal GEE results were still presented for reference, and a partial proportional odds GEE model was further applied to relax covariates that violated the parallel-slope assumption, to validate the robustness and directional trend of the UJD effect.

For the secondary analysis, a linear repeated-measures GEE model was implemented in a long-data format to compare preoperative and postoperative ROM. Measurement time (preoperative = 0, postoperative = 1) was defined as the repeated factor, and the model was adjusted for demographic and surgical covariates. HO grade and the time-by-HO interaction term were further incorporated to evaluate whether HO severity correlated with the magnitude of postoperative ROM change. Stratified Spearman rank correlation analysis was performed separately for the C3–4, C4–5, C5–6, and C6–7 segments only for exploratory subgroup observation. All tests were two-tailed, and *P* < 0.05 was considered statistically significant.

## 3 Results

### 3.1 Imaging reliability assessment

Interobserver consistency analysis yielded a Cohen's Kappa of 0.82 for CT-based UJD grading and 0.78 for CT-detected HO, indicating substantial inter-rater agreement. The Kappa coefficient for HO graded on plain radiographs was 0.75, representing good consistency between observers. Intraobserver ICCs reached 0.91 for UJD and 0.89 for HO, demonstrating excellent repeatability of individual measurements. Minor discrepancies during image evaluation were fully resolved via consensus discussion, with no requirement for third-party adjudication.

### 3.2 Baseline clinical and segmental characteristics

A total of 40 patients with 50 operative segments were enrolled, and there were no missing data for any of the analyzed variables. Demographic and surgical features of patients are summarized in Table 1, and segment distribution, UJD grades, McAfee HO grades, and segmental ROM data are presented in Table 2. The overall incidence of postoperative HO was 92.0%. The mean preoperative segmental ROM was 8.57° ± 4.20°, and the median postoperative ROM was 5.31° (2.05°, 9.30°), with an average ROM reduction of 2.73° ± 5.46°.

**Table 1:** Patient-level baseline characteristics (*n* = 40).

Characteristic	Value
Age, years	49.27 ± 9.30
Sex: male	19 (47.5%)
Sex: female	21 (52.5%)
Follow-up duration, years	1.00 (0.50, 1.81)
Single-level surgery	30 (75.0%)
Two-level surgery	10 (25.0%)

Age is presented as mean ± standard deviation; follow-up duration as median (25th, 75th percentile); categorical variables as *n* (%).

**Table 2:** Operated-segment characteristics and outcome distribution (*n* = 50 segments).

Characteristic	Value
Operated level: C3–4	3 (6.0%)
Operated level: C4–5	17 (34.0%)
Operated level: C5–6	25 (50.0%)
Operated level: C6–7	5 (10.0%)
UJD grade 0	1 (2.0%)
UJD grade 1	10 (20.0%)
UJD grade 2	28 (56.0%)
UJD grade 3	11 (22.0%)
McAfee HO grade 0	4 (8.0%)
McAfee HO grade 1	16 (32.0%)
McAfee HO grade 2	12 (24.0%)
McAfee HO grade 3	13 (26.0%)
McAfee HO grade 4	5 (10.0%)
HO occurrence (McAfee grades 1–4)	46 (92.0%)
Preoperative ROM, °	8.57 ± 4.20
Postoperative ROM, °	5.31 (2.05, 9.30)
ROM loss, °	2.73 ± 5.46

ROM loss = preoperative ROM − postoperative ROM; positive values indicate postoperative loss of segmental motion.

### 3.3 Binary logistic GEE: association between UJD grade and HO occurrence

The binary logistic GEE model, adjusted for age, sex, follow-up duration, number of operative levels, and preoperative ROM, showed that each one-grade increase in UJD was positively associated with higher odds of HO formation, but this trend failed to reach statistical significance (adjusted OR = 12.561, 95% CI 0.885–178.247, *P* = 0.061) (Table 3). Older age [adjusted odds ratio (aOR) = 0.707, *P* = 0.012] and greater preoperative ROM (aOR = 0.601, *P* = 0.013) were independent protective factors against HO (Table 3). Only four segments exhibited no HO, resulting in severely imbalanced outcome distribution and extremely wide confidence intervals for the UJD effect, which implied unstable parameter estimation. Consequently, no independent predictive effect of UJD on HO occurrence could be confirmed.

**Table 3:** Multivariable binary logistic GEE for HO occurrence.

Predictor	B	SE	Adjusted OR	95% CI	*P*
UJD grade (per 1-grade increase)	2.531	1.353	12.561	0.885–178.247	0.061
Age (per 1-year increase)	−0.346	0.138	0.707	0.540–0.927	0.012
Sex (male vs. female)	3.094	2.017	22.075	0.423–1151.010	0.125
Follow-up duration (per 1-year increase)	−0.052	0.392	0.949	0.440–2.046	0.894
Number of operated levels (two vs. single)	0.794	1.511	2.211	0.114–42.750	0.600
Preoperative ROM (per 1° increase)	−0.509	0.204	0.601	0.403–0.898	0.013

Outcome: HO occurrence (0 = McAfee grade 0; 1 = McAfee grades 1–4). The model adjusted for age, sex, follow-up duration, number of operated levels, and preoperative ROM.

### 3.4 Ordinal cumulative logistic GEE: UJD and HO severity

The global proportional odds test was significant (*χ*^2^ = 158.855, df = 18, *P* < 0.001), violating the parallel-slope assumption; age, follow-up duration, and preoperative ROM exhibited threshold-specific effect heterogeneity, whereas UJD grade satisfied the assumption (*P* = 0.31) (Table 4). The primary ordinal GEE showed that each one-grade UJD increase raised the cumulative odds of severe HO by 49.3% without statistical significance (aOR = 1.493, 95% CI 0.863–2.585, *P* = 0.152), and longer follow-up independently predicted higher HO grades (aOR = 1.989, *P* = 0.004) (Table 5). Partial proportional odds GEE sensitivity analysis yielded a consistent positive non-significant association between UJD grade and severe HO (aOR = 1.616, 95% CI 0.905–2.885, *P* = 0.104) (Table 6). Concordant effect directions across both models indicated insufficient evidence that preoperative UJD independently exacerbates postoperative HO severity.

**Table 4:** Proportional odds assumption tests for the ordinal model.

Test	*χ* ^2^	df	*P*
Global test	158.855	18	<0.001
UJD grade	3.134	3	0.371
Age	8.850	3	0.031
Sex	2.503	3	0.475
Follow-up duration	13.406	3	0.004
Number of operated levels	3.243	3	0.356
Preoperative ROM	11.037	3	0.012

Threshold-specific cumulative logistic GEE models were used to assess slope homogeneity across McAfee cut points. *P* < 0.05 indicates evidence against the parallel-slopes/proportional odds assumption.

**Table 5:** Prespecified cumulative ordinal GEE for McAfee HO grade.

Predictor	B	SE	Adjusted OR	95% CI	*P*
UJD grade (per 1-grade increase)	0.401	0.280	1.493	0.863–2.585	0.152
Age (per 1-year increase)	−0.030	0.033	0.970	0.909–1.036	0.366
Sex (male vs. female)	0.142	0.555	1.153	0.389–3.420	0.798
Follow-up duration (per 1-year increase)	0.688	0.237	1.989	1.251–3.163	0.004
Number of operated levels (two vs. single)	0.257	0.681	1.293	0.340–4.913	0.706
Preoperative ROM (per 1° increase)	0.038	0.064	1.039	0.916–1.178	0.549

Outcome: ordered McAfee grades 0–4. An OR >1 indicates higher cumulative odds of being in a more severe HO category. The global proportional odds assumption was violated; results should therefore be interpreted cautiously.

**Table 6:** Partial proportional odds GEE sensitivity analysis for the UJD effect.

Predictor	B	SE	Adjusted OR	95% CI	*P*
UJD grade (per 1-grade increase)	0.480	0.296	1.616	0.905–2.885	0.104

The proportional odds restriction was relaxed for age, follow-up duration, and preoperative ROM, while common slopes were retained for UJD, sex, and number of operated levels.

### 3.5 Linear repeated-measures GEE model for preoperative and postoperative ROM changes

Adjusted linear repeated-measures GEE showed postoperative ROM declined by a mean of 2.732° (*β* = −2.732, 95% CI −4.345 to −1.119, *P* = 0.001), and longer follow-up corresponded to smaller postoperative motion (*β* = −0.685, *P* = 0.001). The interaction of time and HO grade was significant (*β* = −1.855, 95% CI −3.045 to −0.666, *P* = 0.002): each higher HO grade corresponded to an additional 1.86° ROM loss over follow-up. The main HO grade effect was insignificant (*P* = 0.340), meaning that baseline ROM was comparable across HO strata; HO severity only altered the degree of motion decline, and this association cannot be used to infer causality.

### 3.6 Exploratory stratified spearman correlation analysis

Stratified Spearman rank correlation was conducted separately for C3–4, C4–5, C5–6, and C6–7 segments to explore unadjusted bivariate relationships. Significant positive correlations between HO grade and ROM reduction were observed at C5–6 (*ρ* = 0.503, *P* = 0.010) and C6–7 (*ρ* = 0.975, *P* = 0.005) (Table 7). No statistically significant correlations between UJD grade and HO or ROM loss were detected across any of the segment subgroups. Notably, the C3–4 (*n* = 3) and C6–7 (*n* = 5) subgroups had contained very small sample sizes, which raised the risk of type I error. All stratified analyses were exploratory and could not validate the primary study hypothesis.

**Table 7:** Spearman rank correlations stratified by operated cervical level.

Level	n	Association	Spearman *ρ*	*P*
C3–4	3	UJD vs. HO	0.500	0.667
C3–4	3	UJD vs. ROM loss	0.866	0.333
C3–4	3	HO vs. ROM loss	0.866	0.333
C4–5	17	UJD vs. HO	0.145	0.580
C4–5	17	UJD vs. ROM loss	−0.138	0.599
C4–5	17	HO vs. ROM loss	0.275	0.285
C5–6	25	UJD vs. HO	−0.025	0.904
C5–6	25	UJD vs. ROM loss	0.181	0.387
C5–6	25	HO vs. ROM loss	0.503	0.010
C6–7	5	UJD vs. HO	0.730	0.161
C6–7	5	UJD vs. ROM loss	0.632	0.252
C6–7	5	HO vs. ROM loss	0.975	0.005

These subgroup analyses were unadjusted. The C3–4 (*n* = 3) and C6–7 (*n* = 5) strata were extremely small, and multiple exploratory comparisons increase the risk of type I error. Results are hypothesis-generating only and should not be used as primary hypothesis tests.

## 4 Discussion

Published data show that HO rates after artificial cervical disc replacement (ACR) vary widely from 2.4% to 94.1% across different prostheses (16–22). Our cohort demonstrated an extremely high HO prevalence of 92.0%, accompanied by a modest yet statistically significant postoperative reduction in segmental ROM. Unadjusted stratified Spearman analyses detected positive crude correlations between preoperative UJD grade and postoperative HO severity at the C4–5, C5–6, and C6–7 levels. Nevertheless, correlation does not imply causation. After accounting for intrapatient clustering and key confounders via binary and ordinal GEE models, UJD grade exhibited no independent predictive effect on HO incidence or severity, conflicting with univariate subgroup observations.

Biomechanically, progressive UJD disrupts uncovertebral joint congruence and induces chronic aberrant mechanical friction, generating a proinflammatory microenvironment that theoretically predisposes patients to ectopic bone formation (6–9). We initially hypothesized that severe baseline UJD would increase postoperative HO risk, though multivariable-adjusted analyses failed to validate this hypothesis, likely for four main reasons. First, near-complete HO development created a marked ceiling effect with only four HO-free segments available; the inclusion of multiple covariates yielded broad confidence intervals and unstable parameter estimates, substantially diminishing statistical power to detect subtle UJD-mediated effects. Second, this single-center retrospective cohort had a limited overall sample size, and small subgroups at C3–4 and C6–7 undermined the reliability of stratified analyses. Third, residual confounding could not be fully eliminated by the covariates incorporated in our models. Fourth, the true effect of UJD on HO may be inherently modest.

The combination of universal HO formation and collective postoperative ROM loss in our cohort raises a clinically relevant question regarding their direct relationship. Repeated-measures linear GEE confirmed this association via a significant time-by-HO interaction term: each one-grade increase in HO severity corresponded to an additional 1.86° loss of segmental motion, while the main effect of HO grade remained non-significant, indicating comparable baseline ROM across all HO subgroups. Mechanistically, mature periprosthetic heterotopic bone forms rigid osseous bridges that restrict segmental flexion and extension, counteracting the motion-preserving advantage that defines ACR. Unlike cohorts with a low HO burden that maintain satisfactory spinal mobility, the nearly universal HO in our series compromised segmental kinematics globally, which largely explains the uniform postoperative decline in ROM.

No standardized quantitative bony grading scheme for UJD has been established to facilitate cross-cohort radiographic comparisons (11, 21, 22). Plain radiography is limited by projection artifacts and anatomical overlap, whereas magnetic resonance imaging lacks sufficient coronal bony detail for morphological assessment (6–11, 25). CT multiplanar reconstruction delivers superior visualization of osseous anatomy, rendering it optimal for UJD grading. We therefore implemented a modified CT-based Kellgren–Lawrence grading system (14), with reliability testing confirming robust inter- and intraobserver reproducibility to overcome the limitations of conventional UJD assessment modalities.

This work provides a rigorous methodological framework for studies involving multilevel ACR. Segments originating from a single patient are statistically independent, introducing bias when analyzed via conventional parametric or chi-squared tests. We constructed a comprehensive GEE workflow encompassing binary logistic GEE for HO occurrence, ordinal cumulative logistic GEE for HO severity, proportional odds assumption testing, partial proportional odds sensitivity analysis, and interaction-adjusted repeated-measures GEE for longitudinal ROM changes. Our findings also highlight critical statistical limitations arising from outcome saturation and scarce negative events, offering practical guidance for sample size planning in future related investigations.

Several limitations of this study merit acknowledgment. First, the retrospective single-center design, combined with enrollment restricted to patients with complete preoperative CT scans, introduced selection bias. Second, subjective variability persists during manual CT grading despite high interobserver reliability. Third, the relatively short follow-up duration cannot capture long-term progressive changes in HO and UJD. Furthermore, no Grade 4 UJD segments were identified within this cohort, restricting the generalizability of our findings to patients with end-stage uncovertebral arthropathy.

Multilevel cervical surgery is further complicated by segment-level anatomical heterogeneity and varying degrees of preoperative degenerative pathology, which substantially influence surgical planning and postoperative outcomes. Recent work by Gökoğlu et al. highlighted that preoperative segment-specific degenerative status exerts important implications for multilevel cervical surgical decision-making, even for motion-preserving arthroplasty procedures (23, 24). Therefore, preoperative evaluation of local degenerative features such as uncovertebral joint morphology should be carefully integrated when selecting appropriate operative segments and prosthesis candidates.

In summary, after adjustment for intrapatient clustering and confounding variables, preoperative UJD severity could not be confirmed as an independent predictor of postoperative HO incidence or severity in this single-center retrospective cohort. While biologically plausible pathways link UJD to ectopic bone formation, the extremely high HO prevalence resulted in too few HO-negative segments and insufficient statistical power; thus, a subtle true UJD effect cannot be definitively excluded. The observed association between HO severity and postoperative ROM loss aligns with prior published work. Future large-scale, multicenter, long-term cohorts with adequate HO-free cases are required to further interrogate the UJD–HO relationship, and unified quantitative CT-based UJD grading criteria should be developed to refine preoperative radiographic stratification and surgical decision-making for ACR candidates.

## 5 Conclusions

In this single-center retrospective ACR cohort, preoperative uncovertebral joint degeneration grade was not an independent predictor of postoperative HO after adjustment for intrapatient clustering and clinical confounders. Despite plausible biological mechanisms, the 92.0% HO prevalence in our series introduced a ceiling effect and sample size constraints, precluding definitive exclusion of a modest underlying association. Our modified CT-based Kellgren-Lawrence grading demonstrated satisfactory reproducibility for uncovertebral joint degeneration assessment, and the established GEE analytical workflow provides a statistical reference for clustered multilevel cervical arthroplasty datasets.

## Data Availability

The raw data supporting the conclusions of this article will be made available by the authors, without undue reservation.
